# The treatment of severe child aggression (TOSCA) study: Design challenges

**DOI:** 10.1186/1753-2000-5-36

**Published:** 2011-11-10

**Authors:** Cristan A Farmer, L Eugene Arnold, Oscar G Bukstein, Robert L Findling, Kenneth D Gadow, Xiaobai Li, Eric M Butter, Michael G Aman

**Affiliations:** 1 Nisonger Center, Ohio State University, Columbus, USA; 2 Youth and Family Research Program, Western Psychiatric Institute and Clinic, Pittsburgh, USA; 3 Department of Psychiatry, Case Western Reserve University, Cleveland, USA; 4 Department of Psychiatry, Stony Brook University, Stony Brook, USA; 5 Center for Biostatistics, Ohio State University, Columbus, USA

**Keywords:** ADHD, disruptive behavior disorder, stimulant, risperidone, drug trial

## Abstract

**Background:**

Polypharmacy (the concurrent use of more than one psychoactive drug) and other combination interventions are increasingly common for treatment of severe psychiatric problems only partly responsive to monotherapy. This practice and research on it raise scientific, clinical, and ethical issues such as additive side effects, interactions, threshold for adding second drug, appropriate target measures, and (for studies) timing of randomization. One challenging area for treatment is severe child aggression. Commonly-used medications, often in combination, include psychostimulants, antipsychotics, mood stabilizers, and alpha-2 agonists, which vary considerably in terms of perceived safety and efficacy.

**Results:**

In designing our NIMH-funded trial of polypharmacy, we focused attention on the added benefit of a second drug (risperidone) to the effect of the first (stimulant). We selected these two drugs because their associated adverse events might neutralize each other (e.g., sleep delay and appetite decrease from stimulant versus sedation and appetite increase from antipsychotic). Moreover, there was considerable evidence of efficacy for each drug individually for the management of ADHD and child aggression. The study sample comprised children (ages 6-12 years) with both diagnosed ADHD and disruptive behavior disorder (oppositional-defiant or conduct disorder) accompanied by severe physical aggression. In a staged sequence, the medication with the least problematic adverse effects (stimulant) was openly titrated in 3 weeks to optimal effect. Participants whose behavioral symptoms were not normalized received additional double-blind medication, either risperidone or placebo, by random assignment. Thus children whose behavioral symptoms were normalized with stimulant medication were not exposed to an antipsychotic. All families participated in an empirically-supported parent training program for disruptive behavior, so that the actual comparison was stimulant+parent training versus stimulant+antipsychotic+parent training.

**Conclusions:**

We hope that the resolutions of the challenges presented here will be useful to other investigators and facilitate much-needed research on child psychiatric polypharmacy.

**Trial Registration:**

ClinicalTrials.gov NCT00796302

## Background

The Treatment of Severe Child Aggression (TOSCA) study is a four-site NIMH-funded investigation of staged polypharmacy with stimulant and antipsychotic medication with adjunctive behavioral treatment (parent training, PT). Development of the protocol encountered numerous scientific, clinical, and ethical problems that were resolved through iterative review cycles and 7 years of cross-site teleconferences. The solutions, which may be useful to other investigators and clinicians, are presented here in the interests of evaluating this and other forms of polypharmacy.

## Pharmacologic Treatment of ADHD, Disruptive Behavior Disorders, and Aggression

Approximately one half of all referred children with attention-deficit/hyperactivity disorder (ADHD) present with comorbid oppositional-defiant disorder (ODD) or conduct disorder (CD), known collectively as disruptive behavior disorders (DBDs) [1]. Among the many psychosocial interventions for these disorders, PT in behavior management (also known as parent management training) is one of the most effective [2-4]. The literature suggests that such psychosocial interventions are the first line of treatment for a DBD; however, maximal effectiveness is often only achieved over time and may even be ineffective in diminishing high levels of aggression [5]. Data from ADHD studies suggest that concurrent medication may boost the effectiveness of behavioral treatment [6], and in the case of comorbid ADHD and DBD, a combination of medication and behavioral treatment is necessary for an optimal effect [7]. A combination intervention may also result in the use of lower overall doses of medication to achieve the same response associated with monotherapy [8,9].

### Psychopharmacologic Monotherapy

Much research has been devoted to pharmacotherapy for aggressive behavior in prepubertal children in part because it is associated with significant immediate impact in academic and social functioning [10] and considerable psychosocial burden. More troubling is the lack of spontaneous remission and consequent later delinquency, substance abuse, sexual promiscuity, and other psychopathology [11]. Research has focused predominantly on four classes of medication: psychostimulants, antipsychotics, mood stabilizers, and α-2 agonists.

#### Psychostimulants

Psychostimulants, including methylphenidate and amphetamine preparations, are the most commonly-prescribed medications for treating ADHD and boast a large volume of literature supporting their efficacy [12]. Longer-term safety and efficacy has also been demonstrated in multiple investigations [13,14]. Although stimulants have been shown to ameliorate aggressive behavior in children with ADHD, comorbidity with CD is associated with lessened efficacy [15]. For example, a large, rigorous study of psychostimulants in children with both ADHD and CD found that active medication was superior to placebo in reducing symptoms of both disorders, though symptoms of CD at endpoint were not generally normalized [16].

#### Mood Stabilizers

Lithium was the first mood stabilizer shown to be effective in reducing aggressive behavior in children with CD in the 1980s [17-19], though at least one trial found no difference from placebo over two weeks [20]. Methodologically rigorous studies of divalproex found it to be superior to placebo in treating explosive temper and mood lability [21] and CD [22] in adolescents. Both divalproex and lithium require vigilant blood monitoring, which is unappealing to many patients and professionals. Mixed evidence has been found for carbamazepine, which was shown to be effective in a pilot study [23], but a later double-blind, placebo-controlled study failed to demonstrate effectiveness [24]. Overall, certain mood stabilizers may deserve further study, but their potential for serious adverse events (AEs) makes other drug classes more attractive.

#### Alpha-2 Agonists

Alpha-2 agonists (e.g., clonidine and guanfacine) alone are effective in treating ADHD symptoms [25,26], although usually less so than psychostimulants. A pilot study produced preliminary support for clonidine alone or in combination with stimulant for treating ADHD and DBD [27]. In a double-blind, placebo-controlled study, Hazell and Stuart [28] showed a significant effect of clonidine on parent-rated conduct symptoms (but not on teacher ratings of CD). Both guanfacine extended release (XR) and clonidine XR have FDA approval for ADHD [29,30]. The α-2 agonists deserve further study in co-morbid ADHD plus DBD.

#### Atypical Antipsychotics

Atypical antipsychotics are putatively more attractive than conventional antipsychotics due to reduced risk of extrapyramidal side effects. Olanzapine, quetiapine, and aripiprazole all have some evidence to support their efficacy in treating aggressive behavior, but many of the studies are open-label or retrospective chart reviews [31]. Weight gain is the most commonly observed AE with these medications.

In children, risperidone is the most frequently studied of the atypical antipsychotics. The first study of risperidone in children with CD found it to be superior to placebo in reducing parent- and clinician-rated measures of aggressive behavior [32]. Several large, randomized controlled trials have been completed in the intervening decade. Two studies showed risperidone to be superior to placebo in ameliorating hostile and aggressive behavior in children with DBD and subaverage IQ [33,34]. Open-label 48 week follow-ups of both studies found risperidone to be well-tolerated and effective [35,36]. Significant AEs include weight gain and elevated prolactin levels. However, pooled data suggested that risperidone does not negatively impact either growth or sexual maturation in children and adolescents over the long term [37]. Although risperidone has the most positive evidence of the atypical antipsychotics and is the most studied, the possible weight gain (with risk of consequent metabolic syndrome) and increased prolactin levels warrant continued monitoring. It is also worth noting that much of the research on atypical antipsychotics has been carried out in samples with below-average IQ; there are few high-quality data in samples with average IQ.

### Polypharmacy

Despite the well-documented efficacy of stimulant monotherapy, a considerable number of youth with both ADHD and aggression continue to manifest symptoms and impairment [38,39]. A meta-analysis of 28 studies demonstrated that effect sizes vary widely in treatment for aggression in children with ADHD, which leaves a clinical quandary of what to do with non-responders [15].

Polypharmacy is common in most areas of medicine (e.g., cardiology, cancer, or seizure treatment), and is becoming more widespread and respectable in psychiatry despite a previous pejorative connotation. One possible reason for the lag in polypharmacy psychiatric research is a relative lack of interest by drug companies in mental health. In a United States-based survey from the early 2000s, over 40% of child and adolescent psychiatric patients were prescribed two or more medications [40]. Patients with chronic and clinically complex conditions were more likely to receive concomitant pharmacotherapy. In an analysis of annual data from the 1996-2007 United States National Ambulatory Medical Care Surveys, there was an increase in the percentage of pediatric patients who were prescribed medication from at least two psychotropic drug classes [41]. During the 12-year period, multiclass psychotropic treatment rose from 14% of visits to 20%.

Combined pharmacotherapy is often used in patients who demonstrate only partial response to monotherapy, but is also useful in a variety of other contexts, including the treatment of comorbid disorders and as adjunctive therapy associated with adverse events. Combining medications with different mechanisms of action makes conceptual sense in refractory cases, and combinations of drugs with reciprocally neutralizing adverse events (AEs; e.g., blood pressure, pulse, weight, and sedation) are particularly appealing. Nevertheless, despite widespread polypharmacy, there are few published data about controlled polypharmacy or co-administration trials in ADHD [42] and/or DBDs; the available literature is reviewed below.

The different mechanisms of action of psychostimulants and α_2_-adrenoceptor agonists may lead to additive beneficial effects treating ADHD and/or associated problems [26] and has become common clinical practice. The joint use of an α-2 agonist and a psychostimulant for ADHD and DBD has been tentatively endorsed by some treatment guidelines [42,43]. Both guanfacine XR and extended-release clonidine are FDA-approved as both adjunctive therapy and monotherapy for treating ADHD. Several trials have shown alpha-agonist and psychostimulant to be more effective than monotherapy in reducing ADHD symptoms [44-46].

Mood stabilizers have also been considered as adjuncts to psychostimulant treatment. Following an open lead-in of psychostimulant, Blader et al. [47] randomized children and adolescents with ADHD and aggression to an 8-week, double-blind, placebo-controlled trial of divalproex. Remission was observed in nearly 60% of children randomized to divalproex, compared to 15% of the placebo group. Divalproex was associated with higher rates of sadness and insomnia than placebo.

Considerable attention has been given to adjunctive atypical antipsychotic use in children and adolescents [41]. For example, Kronenberger and associates [48] added quetiapine to methylphenidate treatment-resistant adolescents with ADHD, DBD, and aggression. The combination was more effective in control of both ADHD and aggressive symptoms than the methylphenidate alone. Two studies focused largely on the tolerability/safety of using stimulants and atypical antipsychotics. In a naturalistic study, Penzner et al. [49] examined children and adolescents receiving various atypical antipsychotics and concurrent stimulants. At 12 weeks, stimulant co-administration did *not *alter the effects of the antipsychotic on body composition or metabolic indices. However, a similar study found disparate results [50]. Despite the use of low antipsychotic doses, concomitant stimulants, and initially low body mass index *z*-scores, a significant proportion of children treated with combined atypical antipsychotic and stimulant developed one or more criteria for metabolic syndrome. Thus, metabolic and weight gain issues remain of critical importance despite coadministration of psychostimulant.

Although these studies help to frame key issues regarding the use of polypharmacy in treating ADHD with DBD, they also have limitations involving (a) blinded status, (b) randomization, (c) placebo control, (d) sequential titration of medications, and/or (e) longer-term follow-up to ascertain ongoing efficacy and safety of chronic use.

It is a curious fact members of the scientific community have engaged in few broadly-based and vigorous examinations of the merits and liabilities associated with polypharmacy for treating young people with psychiatric problems. Although there are some preliminary data, existing studies are limited in advancing such a discussion. We hope that the following study will help to inform the discussion about the use of a common form of polypharmacy for managing aggression and hostility associated with co-morbid ADHD and DBD.

## Design Challenges

### Final Study Design

**Figure 1:**
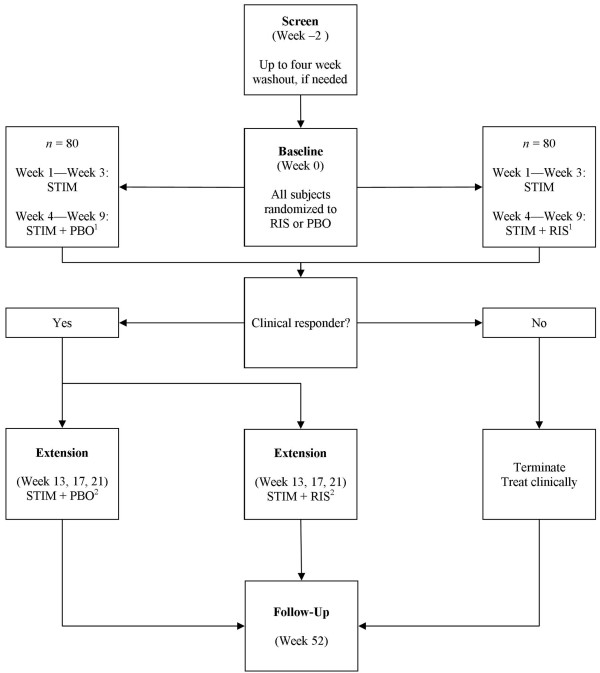
**At screen, arrangements were made to discontinue most medicines for 2 weeks**. Fluoxetine and antipsychotics were washed out for 4 weeks. At baseline, participants were randomized to psychostimulant (STIM; usually OROS methylphenidate) plus placebo or combined (STIM + risperidone [RIS]) treatment. ^1^If subjects did not demonstrate sufficient improvement by end of Week 3 (defined as normative value + 0.5 standard deviation on the NCBRF D-Total score), PBO/RIS was added to treatment. At end of Week 9, subjects were classified as clinical responders (CGI-I = 1 or 2 and NCBRF Disruptive Total reduced by 25% relative to baseline). ^2^Responders were followed on their originally assigned conditions for 12 weeks of double-blinded Extension. Nonresponders were treated clinically as appropriate, based on the study team's best judgment. On the one-year anniversary date from baseline, all participants were asked to return for a follow-up assessment.

Figure 1 shows the final design of the parallel-groups randomized clinical trial. The participants were 160 children aged 6 to 12 years (inclusive), with ADHD, ODD or CD, and significant aggressive behavior. Significant aggressive behavior was defined as a score of three or greater on the Modified Overt Aggression Scale and a score greater than 26 (> 90^th ^percentile) on the Nisonger Child Behavior Checklist Disruptive Total (NCBRF D-Total) subscale. The children were randomized in a 1:1 ratio to two 9-week treatment strategies: stimulant alone for three weeks and, if residual symptoms remained, risperidone or placebo added for six weeks. Dosing for both medications was optimized by weight (see titration schedules in Table 1). Osmotic Release Oral System (OROS) methylphenidate (Concerta) was initiated at 18 mg each morning and titrated up to a limit of 54 mg or 72 mg (depending on weight) by Week 2. Risperidone was initiated at 0.5 mg each evening and titrated up to a total daily limit of 2.5 mg or 3.5 mg, depending on weight. Dosage was held at a lower level or reduced if limiting side effects developed or if clinical improvement left no meaningful room for further improvement. To be consistent with recommended practice and to keep the study ecologically valid, the parents/guardians of all participants received parent training (PT) in strategies of behavior management. The double-blind was maintained in a three-month extension for those deemed responders (CGI-I of 1 or 2, plus greater than 25% reduction in NCBRF D-Total) at Week 9. This extension provided the opportunity to observe whether the short-term effects were maintained over the medium-term. Finally, a one-year follow-up (Week 52) was sought for all participants, in order to assess feasibility, efficacy, and safety over the long term.

**Table 1:** Dosing and Titration Guidelines

	**OROS Methylphenidate**		**Risperidone**				
	
**Day**	**< 25 kg**	**> 25 kg**	**Day**	**20-45 kg**		**> 45 kg**	
		
				**AM**	**PM**	**AM**	**PM**
					
1-4	18 mg	18 mg	1	-	0.5 mg	-	0.5 mg
5-7	18 mg	36 mg	4	0.5 mg	0.5 mg	0.5 mg	0.5 mg
8-11	36 mg	54 mg	8	0.5 mg	1.0 mg	0.5 mg	1.0 mg
12-14	54 mg	72 mg	11	-	-	1.0 mg	1.0 mg
15+	Maintenance; Dose reduction allowed for side effects		15	-	-	1.0 mg	1.5 mg
			16	1.0 mg	1.0 mg	-	-
			18	-	-	1.5 mg	1.5 mg
			22	1.0 mg	1.5 mg	1.5 mg	2.0 mg
			29	1.0 mg	1.5 mg	1.5 mg	2.0 mg

The study prescriber could vary from the guidelines as clinically indicated. In particular, the dose could be held constant or reduced for limiting side effects or if the clinical status left no room for further improvement.

### Trial Design Challenges

One challenging design issue was the selection of possible treatment arms. Beyond a simple placebo arm, there were several options under consideration, including: (a) random assignment to three conditions (each drug alone and their combination); (b) random assignment to two conditions, one drug alone or to both drugs; (c) random assignment to drug A with drug B to be added or the reverse; and (d) open-label administration of one drug with a randomly-assigned second drug (or placebo) to be added *if needed *at a predefined time with a predefined threshold. The first three options required exposure to both drugs by random assignment rather than demonstrated need. This was an ethical concern recognized by both the TOSCA investigators and the NIH review committee, and we therefore adopted the fourth option. We decided first to try the stimulant owing to its more favorable AE record and then to add the antipsychotic if needed. In order to remain in the stimulant-only condition, the child had to obtain a score within one-half standard deviation of the mean for a normative sample on the main outcome measure of ODD behaviors (including aggression; NCBRF D-Total) *and *a CGI Improvement rating of "Very much improved."

#### Timing of Randomization

A related issue pertained to the timing of randomization, which was partially determined by the review process. Once we decided the stimulant medication should be openly titrated and the second medicine (risperidone) added if needed, we proposed to randomize less-than-optimal stimulant responders to either risperidone or placebo for six weeks. This strategy seemed cleanest, with the intent-to-treat (ITT) analyses confined to stimulant *poor responders*, but the NIMH review committee was concerned about attrition before the randomization. Therefore, we decided to randomize enrolled subjects at baseline to the two treatment strategies: stimulant plus placebo versus stimulant plus risperidone. However, only those participants who evidenced less-than-optimal response to stimulant were actually given the second medication (placebo or risperidone). Details on the timing of second medication are discussed in a later section. In retrospect this strategy appears to have been unnecessary, as we had relatively little attrition prior to randomization. However, our adopted strategy had two related advantages: it is compatible with (though not identical to) the choices that a prescriber faces, and it directly answers the question of whether anticipating combined drug therapy from the beginning is a good treatment strategy.

#### Placebo or not?

An additional consideration we faced was the inclusion of a placebo arm. Ideally, it would be optimal to compare groups of children treated with each drug separately and their combination to a fourth group who received placebo only. A two-by-two design could accomplish this, using a double-placebo with all participants taking two kinds of pills, each of which could be either placebo or active. However this design would prescribe polypharmacy as a result of randomization rather than clinical need, leaving us with ethical reservations about exposing some patients to the added risk of the second drug unnecessarily. Conversely, because numerous well-controlled studies had established the efficacy of both drugs versus placebo, it could have been argued that a placebo-alone arm may have unnecessarily increased the cost of the study as well as delayed treatment for patients deemed sick enough to benefit from the combination of two drugs. Ultimately, the practical realities of conducting such a study determined the course of action. The funds that NIMH could afford to invest in TOSCA would not accommodate a placebo arm. In retrospect, this was fortunate, as many of the highly stressed and often fragile families who qualified for TOSCA have encountered difficulty adhering to treatment *known *to be active and would likely have found it impossible to weather a placebo assignment. Another benefit was incurred during recruitment because we were able to inform potential participants that everyone would get two treatments (PT and stimulant) and half would be randomized to get a third treatment if needed.

#### Selection of Psychosocial Treatment

The selection of concurrent PT was driven by two important factors. The most pressing was the desire to provide active therapy to participants randomized to the stimulant plus placebo arm. Also important, however, was the notion that federally-funded research is charged with examining treatments using best clinical practices. Owing to powerful cultural beliefs that DBDs are more environmentally driven than CNS diseases such as schizophrenia [51], we concluded that youth with aggression should be treated first with behavioral intervention prior to the administration of antipsychotic medication. Ultimately, this propelled us into a design that offered the best currently available interventions (PT and psychotropic medication).

We opted to use a conventional behavioral parent training approach that emphasized the basics of positive reinforcement, planned ignoring, promoting positive transitions, incentive systems, collaborative parent-child planning, and time-out [52]. The approach used, an adaptation of Community Parent Education (COPE), provided for individual application to each family and child regardless of age, clinician modeling of the intervention, and parent-clinician role play practice of the intervention as applied to each child.

### Medication Selection

Medication selection was based in large part on the following: prior research demonstrating the efficacy of each drug's monotherapy, actual prescribing patterns in the United Sates (including polypharmacy), evidence for acceptability, and the possibility of beneficial interactive effects, both therapeutic and untoward, owing to diverse mechanisms of actions. Our initial choice was divalproex because of its common clinical use in the target population. However, in two consecutive submissions, individual reviewers were adamantly opposed to the choice of divalproex, citing adverse events and limited data supporting efficacy.

It was fortuitous that two of the most commonly prescribed drugs for child aggression may have potentially offsetting AEs. Stimulants may depress appetite, interfere with sleep, and induce rebound irritability (as they wear off at the end of the day). Conversely, antipsychotics may increase appetite, cause sedation, and have sufficiently long half-life to reduce the risk of withdrawal effects when treatment is abruptly terminated. This seemed to offer reasonable hope that the combination may be associated with fewer side effects than either alone; one aim of the study was to assess AEs as well as additive benefit. Additionally, the soaring popularity of atypical agents and attendant concerns of advocacy groups calling for more controlled trials in children made risperidone an obvious choice. Thus, we settled upon treatment with psychostimulant followed, if necessary, by risperidone versus placebo.

Although the stimulant of first choice in this trial was OROS methylphenidate owing primarily to its extended duration of action, it was not mandated in the protocol so as not to exclude children who had prior poor response or who had difficulty swallowing pills. It was required that the substituted medication be a stimulant because the addition of non-stimulant ADHD medications would have introduced "noise" into the analyses. In such cases, the dosage was matched in potency to the OROS methylphenidate taken by most other participants. This was consistent with common clinical practice in the United States, where a tolerable stimulant would ordinarily be sought before adding an antipsychotic.

#### Timing of Second Medication

It was necessary to decide how long to allow for an effect of stimulant before adding the second medication. The literature suggests the treatment effects of a given dose are observed within a day after initial administration [53]. Therefore, we decided that stimulant monotherapy could be titrated within two weeks by making use of mid-week adjustments and allowing a third week to observe the effect of the optimal dose. At the end of Week 3 stimulant response was assessed and a decision made about adding the second medication. However, if the second drug was not needed at Week 3 and the child's behavior subsequently deteriorated on the optimal stimulant dose, the prescriber was able to add the second medication through the sixth week of the study. This would allow at least three weeks for detection of any effect that the second medication might have. This rescue policy was important because an ITT analysis was proposed to analyze the data from each child within the group to which (s)he was *randomized*, regardless of whether or not the second medication was actually taken. Therefore, constraining the addition of the second medication exclusively to the Week 3 visit would have had deleterious effects on our ability to show an additive effect, as well as doing the child a clinical disservice.

### Instrumentation

An important component of any study is the selection of appropriate measures. In the current study, it was necessary to assess multiple behavioral constructs. Additionally, the instruments used to assess AEs were required to span several categories relevant to both stimulant and antipsychotic medications. Here we present the rationale for the selection of our main study instruments.

#### Primary and Secondary Outcomes

Table 2 contains an abbreviated schedule of measures constrained to those relevant to polypharmacy issues. The main behavioral constructs of interest were symptoms of ADHD, ODD/CD, and aggression. To that end, the primary outcome measure was the parent-completed NCBRF Disruptive-Total score. The NCBRF [54] for typically-developing children has one Prosocial subscale and six Problem Behavior subscales (Conduct Problem, Oppositional Behavior, Hyperactive, Inattentive, Overly Sensitive, and Withdrawn/Dysphoric). The D-Total is the sum of the Conduct Problem and Oppositional Behavior subscales. We felt that it represented the range of behaviors expected to improve with the combined treatment (i.e., pharmacological *and *behavioral). It has excellent psychometric characteristics and, based on findings with the developmental delay version in samples of children with cognitive delay [33], we expected it to be highly treatment-sensitive.

**Table 2:** Abbreviated Schedule of Measures

Measure or Procedure	Screen	W0	W1-2	W3	W4-8	W9	W13-17	W21	W52
***Diagnostic Assessments (Screen only)***									
Kiddie Schedule for Affective Disorders	**X**								
General Behavior Inventory	**X**								
***Medical Clinician***									
Blood draw	**X**					**X**		**X**	**X**
Vitals, Height, Weight, AEs	**X**	**X**	**X**	**X**	**X**	**X**	**X**	**X**	**X**
Concomitant Medications	**X**	**X**	**X**	**X**	**X**	**X**	**X**	**X**	**X**
***Blinded Clinician***									
Family Assessment Device	**X**								
Modified Overt Aggression Scale (Inclusion)	**X**								
Antisocial Behavior Scale		**X**				**X**			**X**
Standardized Observation Analogue Procedure		**X**				**X**			**X**
Child Symptom Inventory-4R	**X**					**X**			**X**
Symptom Checklist-4	**X**	**X**	**X**	**X**	**X**	**X**	**X**	**X**	**X**
Nisonger Child Behavior Rating Form	**X**	**X**		**X**	**X**	**X**	**X**	**X**	**X**
Clinical Global Impressions-Improvement		**X**	**X**	**X**	**X**	**X**	**X**	**X**	**X**
Clinical Global Impressions-Severity	**X**	**X**		**X**		**X**		**X**	**X**
***Teacher Ratings***									
Antisocial Behavior Scale		**X**				**X**			**X**
Child Symptom Inventory-4R		**X**				**X**			**X**
Symptom Checklist-4		**X**	**X**	**X**	**X**	**X**	**X**	**X**	**X**
Stimulant Side Effects		**X**	**X**	**X**	**X**	**X**	**X**	**X**	**X**

Cells marked with an **X **indicate visits in which the assessment was performed.

Secondary measures included the Clinical Global Impressions Scale (CGI) [55] Severity and Improvement, and the Standard Observation Analogue Procedure (SOAP) [56]. The Standard Observation Analogue Procedure (SOAP) is a direct observation procedure that provides an objective video record of caregiver-child interactions for purposes of assessing caregiver skill acquisition and child response to intervention. The SOAP was included to provide an objective index (coders are blinded to treatment assignment) that can be used to verify parent/teacher/clinician ratings of behavior. We also anticipated that the SOAP might be helpful in assessing change in *parental *behavior as a result of PT.

There is some evidence to suggest that certain subtypes of aggressive behavior might be more responsive to pharmacologic treatment [57]. Thus, proactive and reactive aggression are assessed with the Antisocial Behavior Scale (ABS) [58], which contains a Proactive Aggression subscale (five proactive items and five covert antisocial items) and a Reactive Aggression subscale (six items). For a similar reason (i.e., potential moderation), the short version (27 items) of the MacMaster Family Assessment Device [59] was used to characterize the family system, which is often chaotic in this study population and might influence response to treatment.

#### Other Measures

The Modified Overt Aggression Scale (M-OAS) [60] has seven items: verbal assault; assaults against objects, against others, against self; global subjective irritability; global overt irritability, and suicidal tendencies. This study made use of three items: (a) assault against objects, (b) assault against others, and (c) assault against self. Each of the items is rated on a scale from zero (no events) to five, where a higher number indicates greater severity. We created an additional selection ("3b") wherein the rating is chosen if the child *would have *completed the behavior, but was prevented by the parent or caregiver; this rating was especially useful for younger children, whose behavior is perhaps more easily contained by parental intervention. The M-OAS has been used in a number of descriptive and pharmacological trials, but it has been used more in adults than for young persons. Thus, it was used in this study only as an *entry *threshold criterion (a score of three or greater on one or more of the items). This degree of aggression was considered ethically necessary to justify the risk of antipsychotic drug.

The population from which this study drew is diagnostically complex. The behaviors observed in children with ADHD and DBD are often similar to those found in mood disorders. Psychosis, mood disorder, and major depression were exclusionary in this study. We screened for these disorders in two ways: The Kiddie Schedule for Affective Disorders and Schizophrenia [61,62], a semi-structured diagnostic instrument administered to both parent/guardian and child by a trained interviewer with clinical experience, was the primary source of diagnostic information. A second screen for the presence of mood disorder was the parent-rated using the General Behavior Inventory [63].

The Child and Adolescent Symptom Inventory-4R (CASI-4R) [64] is a 147-item parent- and teacher-completed rating scale for evaluating youth 5 to 18 years. Individual items bear one-to-one correspondence with DSM-IV symptoms. Therefore, parent and teacher reports on the ADHD and Peer Conflict scales (known together as the ADHD Symptom Checklist-4) [65] were assessed at every study visit. In addition, the full CASI-4R was collected at key points in the study (screen, baseline, and endpoints). The CASI-4R subscales were a source of potential moderators of treatment response and helped us to detect pharmacological effects on a wide array of outcomes that might not otherwise be covered.

#### Teacher Ratings

Although obtaining information from school personnel about therapeutic response and untoward reactions is for many an essential element in a best practices design, this can also create many challenges for longer-term designs. Limiting the completion of the full study to coincide with the school year would greatly restrict recruitment and increase cost burden. Our solution to this situation was to obtain diagnostic-related information (see Table 2) from the current teacher. During the clinical trial, ratings were obtained from teachers for all assessment periods for which school was in session, making accommodations for school holidays when necessary. This method of combining ratings from different sources is likely suboptimal; however, in some cases, this was the only viable alternative.

#### Adverse Events/Safety Assessment

Safety is a particularly important and understudied issue in polypharmacy. Due to the use of two separate drug classes, it was necessary to assess several areas of potential adverse events. A specific emphasis was placed on extrapyramidal symptoms, appetite, weight, sleep patterns, and metabolic aberrations, as these AEs are commonly associated with the study drugs. Two parent report AE scales were used; one specific to stimulants, the other specific to antipsychotics. Both were based on AEs listed in package inserts and were used in previous studies. Treatment-induced motor disturbances were assessed with three clinician-completed measures. The Abnormal Involuntary Movement Scale (AIMS) [55] is a standardized clinician-rated review of tremor, dyskinesia, and other "active" neuromotor side effects. The Simpson-Angus Rating Scale [66] complements the AIMS in checking for "passive" extrapyramidal side effects (rigidity, dystonia, and abnormal glabellar reflex). The Barnes Akathisia Scale [67] is a four-item scale with two items based on observation by the rater and two items reflecting the patient's experience of restlessness.

Clinical laboratory tests checked for metabolic effects of the antipsychotic, including complete blood count, liver enzymes, lipids, fasting glucose, hemoglobin A1c, and prolactin. All participants were administered an electrocardiogram at screen and endpoint. Vital signs included heart rate and blood pressure, possibly affected by both drugs. Given the prominence of weight change with both medications, height, weight, and hip-waist ratio were also collected.

### Statistical Considerations

In a two-drug additive design the primary analysis should measure the effect of the second drug when added to the first. This could be done in two ways. One would be to take a new baseline before the second drug is added and analyze the change score from the new baseline to endpoint between those receiving placebo and those receiving the second drug. Although this has the advantage of clearly focusing on additive effect, it is most appropriate if randomization occurs at the point of adding the second drug. Because we randomized before starting the first drug, we were comparing the monotherapy strategy to the strategy of adding a second drug if needed. Therefore we primarily compared the change scores in NCBRF D-total from Baseline to Week 9 between the two treatment strategies. Data from all randomized subjects were included based on ITT principles. Linear mixed effects models were used to analyze the repeatedly measured outcomes.

A preliminary statistical challenge was the power analysis, which had to be based on the expected additive effect. The additive effect can be quite different from the placebo-controlled effect size (ES) of either single drug. Further, the ES can be diminished by the concomitant parent training provided for ethical and recruitment reasons in this study. Aman, Binder, and Turgay [68] showed an ES of Cohen's *d *= 0.81 when risperidone was added to pre-existing stimulant without PMT, but the stimulant was neither given prospectively nor was the dose optimized in that study. Taking into account the effect of PMT, we conservatively estimated an ES of Cohen's *d *= 0.5 for the difference between treatment with one and two drugs, where there would be a difference of 5.5 and a common standard deviation of 11 in the NCBRF D-Total change scores between the two treatment groups. With complete data on 124 subjects (64 per group), we would be able to detect such an ES with 80% power using a two-sample *t*-test at a two-sided type I error rate of 0.05. To allow for a possible 20% dropout rate prior to the second drug, we recruited about 80 subjects per group. Randomization at baseline was blocked on site and diagnosis (ODD vs. CD) to allow for post-hoc tests of differential treatment effects. Linear mixed effects models can handle missing data based on the untestable assumption that unobserved values are missing at random, though efforts were made to minimize missing data during the trial. Last-observation-carried-forward was used to check the sensitivity of the primary analysis to the assumption that data were missing at random.

### Study Monitoring

The study design developed for TOSCA also posed unique challenges for cross-site study monitoring. As in any medication trial, it was important to insure that the titration protocol was followed uniformly across sites for both medications. In this regard, it was particularly challenging to ensure that the first medication was adequately titrated before the second medication was added, because the protocol prohibited dose increases of stimulant following the addition of risperidone/placebo.

The multiple decision points in this study dictated different definitions of "responder." At the Week 3 visit, children with a CGI-I of 1 and a NCBRF D-Total score below 15 (e.g., within 0.5 SD of the normative mean) were considered superb responders to stimulant, not requiring the second medication; by protocol, others were to receive the second drug. Our rationale was that we wanted to test the advantage of the second medication if there was room for improvement. A different definition of "responder" was used at the Week 9 decision point. Children with a CGI-I of 1 or 2 and a NCBRF D-Total score that was improved at least 25% relative to baseline were considered responders at Week 9 and were enrolled in the three-month Extension; others exited the study to personalized clinical care. These different definitions of clinical responder added a degree of complexity not usually found in drug studies. Thus cross-site fidelity monitoring was needed to verify that the two different definitions of responder were applied at their appropriate decision points.

## Clinical Significance

The use of polypharmacy to treat disruptive behavior disorders and ADHD has become standard practice in the United States without adequate pertinent data. The existing literature provides some support, but also suggests concerns. Rigorous studies of polypharmacy are complicated by scientific, ethical, and practical issues. Scientific challenges include the choice of drugs, dosing and timing of the combination, assessment of drug effects, and the advisability of a placebo. Ethical concerns regarding the possible unnecessary addition of a second drug or the lack of a necessary addition (i.e., placebo) in severe cases are important considerations in the trial design. Finally, practical challenges such as the additional expense of a second medication, the concern of regulatory bodies such as institutional review boards or data safety monitoring boards regarding polypharmacy, and the administration of several treatments concomitantly are unique to polypharmacy studies. We hope that the resolutions of these challenges presented here will be useful to other investigators and facilitate much-needed research on polypharmacy.

## Competing interests

Dr. Aman receives or received research support, acted as a consultant, and/or served on a speaker's board for Bristol-Myers Squibb, Research support; Johnson & Johnson, research support; Hoffman La Roche, Advisory Board; BioMarin Pharmaceutica, Advisory Board; Forest Research, Advisory Board, research support. Dr. Arnold receives or received research support, acted as a consultant, and/or served on a speaker's board for Lilly, Shire, Curemark, Novartis, Targacept, AstraZeneca, Noven, and Seaside Therapeutics. Dr. Bukstein receives royalties from Guilford Press and Routledge. He also receives research support from Shire Pharmaceuticals. Dr. Findling receives or has received research support, acted as a consultant and/or served on a speaker's bureau for Abbott, Addrenex, Alexza, AstraZeneca, Biovail, Bristol-Myers Squibb, Forest, GlaxoSmithKline, Johnson & Johnson, KemPharm Lilly, Lundbeck, Merck, Neuropharm, Novartis, Noven, Organon, Otsuka, Pfizer, Rhodes Pharmaceuticals, Sanofi-Aventis, Schering-Plough, Seaside Therapeutics, Sepracore, Shire, Solvay, Sunovion, Supernus Pharmaceuticals, Validus, and Wyeth. Dr. Gadow is a shareholder of Checkmate Plus, publisher of the Child and Adolescent Symptom Inventory-4R.

## Authors' contributions

CAF and LEA drafted the manuscript, which all authors contributed to and approved of. LEA, MGA, OGB, RLF, and KDG conceived of and participated in the design of the study. XL provided statistical consultation.
